# Costs and benefits of a subtype-specific surveillance system for identifying Escherichia coli O157:H7 outbreaks.

**DOI:** 10.3201/eid0603.000310

**Published:** 2000

**Authors:** E H Elbasha, T D Fitzsimmons, M I Meltzer

**Affiliations:** Centers for Disease Control and Prevention, Atlanta, GA 30341, USA. ebe4@cdc.gov

## Abstract

We assessed the societal costs and benefits of a subtype-specific surveillance system for identifying outbreak-associated Escherichia coli O157:H7 infections. Using data from Colorado, we estimated that if it averted five cases annually, the system would recover all its costs.


### Costs and Benefits of a Subtype-Specific Surveillance System for Identifying *Escherichia coli* O157:H7 Outbreaks

**Elamin H. Elbasha,* Thomas D. Fitzsimmons,*† and Martin I. Meltzer***
*Centers for Disease Control and Prevention, Atlanta, Georgia, USA; and †Colorado Department of Public Health and Environment, Denver, Colorado, USA

We assessed the societal costs and benefits of a subtype-specific surveillance system for identifying outbreak-associated ***Escherichia coli*** O157:H7 infections. Using data from Colorado, we estimated that if it averted five cases annually, the system would recover all its costs.

***Escherichia coli*** O157:H7 infections pose a serious public health threat (1-4). Surveillance, rapid reporting of cases, and prompt epidemiologic investigations are essential elements of timely public health response (2,5). Surveillance that uses molecular subtyping methods has at least two advantages over traditional surveillance systems (6). First, it is sensitive enough to identify outbreaks not detected by traditional surveillance or can detect them earlier. Second, it is specific enough to differentiate sporadic cases from outbreak-related cases and distinguish between single and multiple outbreaks.

A subtype-specific surveillance system consists of 1) mandatory submission of ***E. coli*** O157:H7 isolates for subtyping; 2) a centrally located laboratory equipped to perform subtyping by pulsed-field gel electrophoresis; 3) active links between local and state health officials; and 4) epidemiologic capacity to investigate the possibility of an outbreak once identical strains are identified.

In August 1997, the Colorado Department of Public Health and Environment, using subtype-specific surveillance, identified an outbreak associated with eating hamburgers from beef processed in a plant in Nebraska and distributed nationally. After the outbreak was traced to the contaminated beef, the company recalled 25 million pounds of ground beef, the largest meat recall recorded (7).

We used cost-benefit analysis to assess the economic feasibility (from a societal perspective) of using a system similar to the one in Colorado for identifying ***E. coli*** O157:H7 outbreaks.

### The Study

A system is cost-beneficial if the discounted benefits it generates are at least as great as the discounted costs of installing and operating the system. The life span of the subtype-specific surveillance system in Colorado is 5 years, yielding benefits over the lifetime of people affected by it. Data on the costs of the system were obtained from the Colorado Department of Public Health and Environment (Table 1). The system was not set up only to subtype for ***E. coli*** O157:H7 but also to identify outbreaks of other organisms (e.g., ***Salmonella typhi***); only ***E. coli***-related costs were considered here. The sensitivity of the results was examined with all the costs of the system attributed to ***E. coli*** O157:H7 subtyping.

In estimating the costs of outbreak investigations, we assumed that, as a result of the system, two epidemiologic investigations would be carried out each year, with an average cost of $9,600 per outbreak (Table 1). The costs associated with recalling any outbreak-related

Address for correspondence: Elamin H. Elbasha, Centers for Disease Control and Prevention, 4770 Buford Highway, Mail Stop K73, Atlanta, GA 30341, USA; fax: 770-488-8464; e-mail: ebe4@cdc.gov.

Table 1. Costs of installing and operating the subtype-specific surveillance system, Colorado, 1996

| Labor and equipment costs | Total costs | ***Escherichia coli***-related costs[a] |
|---|---|---|
| Equipment | $40,000 | $16,000 |
| Laboratory scientist (per year)[b] | $10,000 | $4,000 |
| Analyzing the isolates (per year)[c] | $12,000 | $12,000 |
| Investigating an outbreak[d,e] | $9,600 | $9,600 |
| Present value of outbreak costs (in 5 years)[f] | $90,568 | $90,568 |
| Annual operating costs[g] | $41,200 | $35,200 |

[a]From the proportion of ***E. coli*** isolates among the total number of isolates expected to be subtyped each year, we extrapolated that 40% of the equipment and labor costs were ***E. coli***-related.
[b]The salary and fringe benefits of a full-time laboratory analyst.
[c]Analyzing 300 isolates at a cost of $40 per isolate.
[d]This cost included, but was not limited to, the value of time (15 days) spent investigating an outbreak, answering telephone calls, conducting meetings, improving and transferring pulsed-field gel electrophoresis image files to various groups, creating databases, requesting information, responding to media calls, and handling legal issues. We assumed that, as a result of the system, two outbreaks would be investigated each year (6).
[e]The costs of additional labor and the epidemiologic investigation of an outbreak were estimated at $5,000 and $4,600, respectively.
[f]At a discount rate of 3%.
[g]Laboratory scientist ($10,000) + analyzing the isolates ($12,000) + investigating two outbreaks (2 x $9,600 = $19,200)

product were not included. Data (e.g., percentage of contaminated beef) that would allow us to attribute economic value to the amount of the product recalled were not available. In the sensitivity analysis, costs were increased by 100% to account for such missing data.

The benefits of the surveillance system are the economic savings accrued from ***E. coli*** O157:H7 cases averted. Determining the number of cases averted as a result of using the system is difficult. One way of determining this number is by estimating the attack rate and multiplying that number by the amount of beef recalled (8).

However, for the outbreak in Colorado, data for estimating specific attack rates were lacking. Instead, we estimated two threshold numbers of cases that must be averted for the costs to be equal to the benefits of the system. The first threshold number was calculated by assuming the system averts a constant number of cases every year. The second number was calculated under the assumption that the system averts only a given number of cases in the first year and no cases in subsequent years. If the estimated threshold is below a reasonable number, the system is cost beneficial. A reasonable number is calculated by consulting the literature and expert opinion.

The average cost of an ***E. coli*** O157:H7 infection was estimated by using an infection outcome tree (4) (Figure). A person infected with ***E. coli*** O157:H7 can be in only one disease severity category (Figure; Table 2). The far-left branch of the tree is designated as severity category no. 9. The probability is 0.2% (10% hospitalization x 50% hemolytic uremic syndrome [HUS] x 4% death) that an infected person will be hospitalized for hemorrhagic colitis, come down with HUS, and die after 1 year. From the time of infection until the time of death, the societal costs for this patient are $991,221 (medical costs $39,204 + productivity losses $3,041 + lost lifetime earnings $948,976).

Data on the probability of being in any of these categories were obtained from Roberts et al. (3). The economic costs associated with each category were based on the methods and assumptions of Buzby et al. (4), with modifications (Table 2). Productivity losses were estimated by multiplying the average wage in 1996 by the number of days missed from work. The average wage rate was estimated by using the average daily earnings of a nonagricultural nonsupervisory employee, assuming that fringe benefits are 39% of total wages or salaries and a labor participation rate of 84% (4). We estimated the costs of a death by using only the lost lifetime earnings, as estimated by Haddix et al. (9) and updated by the rates of change in wages. Because we did not assess pain and suffering from the disease or loss of human life, our estimates of benefits should be considered conservatively low.

All costs and benefits were adjusted to 1996 dollars according to the consumer price index or its components (various issues of the Statistical Abstract). All future costs and benefits were

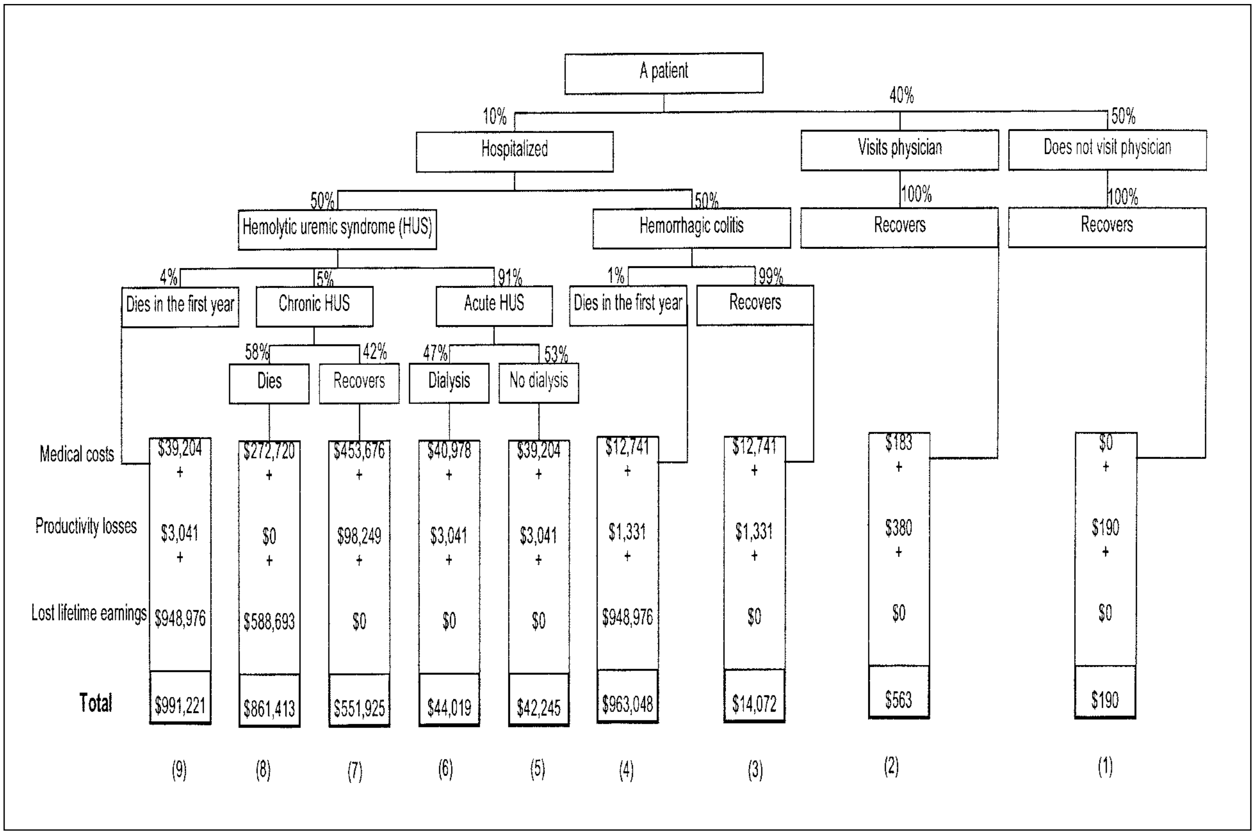

Figure. ***Escherichia coli*** O157:H7 infection outcome tree. Severity categories (1)-(9) are described in Table 2.

Table 2. Assumptions about disease severity following an ***Escherichia coli*** O157:H7 infection[a]

| Severity category | Assumptions |
|---|---|
| No. 1 | Patient does not seek medical care, recovers, and misses 2 days of work |
| No. 2 | Patient seeks medical care for hemorrhagic colitis, has one laboratory test, recovers, and misses 4 days of work |
| No. 3 | Patient is hospitalized for hemorrhagic colitis for 6.5 days and recovers after missing 14 days of work |
| No. 4 | Patient is hospitalized for hemorrhagic colitis for 6.5 days, misses 14 days of work, and dies in the first year |
| No. 5 | Patient is hospitalized for acute HUS[b] for 5 days in ICU[c] and 10 days in a regular room, and recovers after missing 32 days of work |
| No. 6 | Patient is hospitalized for acute HUS[b] for 5 days in ICU[c] and 10 days in a regular room, requires dialysis for 12 days, and recovers after missing 32 days of work |
| No. 7 | Patient is hospitalized for hemorrhagic colitis; comes down with chronic HUS[b]; may require dialysis, transplants, or drug therapy; cannot work for an extended period; and recovers |
| No. 8 | Patient is hospitalized for hemorrhagic colitis; comes down with chronic HUS[b]; may require dialysis, transplants, or drug therapy; cannot work for an extended period; and dies |
| No. 9 | Patient is hospitalized for acute HUS[b] for 5 days in ICU[c] and 10 days in a regular room and dies after missing 32 days of work |

[a]Adapted from Buzby et al. (4). A patient is defined as a person infected with ***E. coli*** O157:H7 who has at least a gastrointestinal illness for more than 1 day.
[b]HUS, hemolytic uremic syndrome.
[c]ICU, intensive care unit.

discounted at 3%. Other rates were used in the sensitivity analysis.

The discounted average cost of an ***E. coli*** O157:H7 infection was $7,788 (Table 3). The main component of the cost of a case was the expected cost of sequelae and death. The undiscounted cost of a case was $15,927. The discounted cost of installing and operating the surveillance system over a period of 5 years was $182,042 (Table 3). Included in this category were the costs of investigating outbreaks ($90,568 in 5 years), of the subtyping equipment ($16,000), and of analyzing the isolates ($60,000 in 5 years) (Table 1). At a 3% discount, five cases per year (or 14 cases in the first year only) must be averted for the costs of the system to be equal to its benefits (Table 3). Without discounting, the threshold number dropped to 2.4 cases per year (Table 3).

### Sensitivity Analysis

The estimated costs of a case were sensitive to the estimates of the probability of death after infection. If the probability of death is raised to 2.3% (4), the cost of a case increases to $25,997, and the threshold number of cases averted for the system to be economically feasible decreases to 1.5 per year for 5 years, or 4.3 cases in the first year and none in the following years (Table 3).

If all the costs of subtyping (including subtyping for other organisms) were included in the analysis, the system would recover its costs after averting 6.4 infections annually, or 20.9 cases in the first year only, with no cases detected in subsequent years. If the costs of the system doubled or the benefits of a case averted decreased by 50%, the threshold number would increase to 9.9 cases per year, or 28.4 cases in the first year only (Table 3). Doubling the number of outbreaks or considering only direct medical costs would raise the threshold numbers to 7.4 and 11.4 cases per year, respectively.

### Conclusions

If 15 cases were averted by the recall of the 25 million pounds of potentially contaminated beef, the Colorado system would have recovered all costs for the 5 years of start-up and operation by detecting a single outbreak (Table 3). In comparison, the outbreak-related 1993 recall of 255,000 regular (0.1-lb) hamburgers in Washington State was estimated to have prevented 800 cases (8).

The discounted average cost of an ***E. coli*** O157:H7 infection of $7,788 (Table 3) was a relatively conservative estimate compared with that of $38,000 (in 1995 dollars) by Buzby et al. (4). The major differences are the probability of death and the economic value of life used in the estimation (11).

If other benefits of the system (e.g., obviating the need to investigate sporadic cases) are included, the system becomes even more cost

Table 3. Discounted costs of an ***Escherichia coli*** O157:H7 infection, discounted costs of the surveillance system, and threshold number of cases, 1996

| | Discount rate[a] | | |
|---|---|---|---|
| Components affecting costs | 3% | 0% | 5% |
| Discounted average cost of an ***E. coli*** O157:H7 infection | $7,788 | $15,927 | $5,847 |
| Discounted costs of installing and operating the system | $182,042 | $192,000 | $176,018 |
| Baseline (best estimate) | | | |
| Cases that need to be averted every year for 5 years[b] | 5.0 | 2.4 | 6.6 |
| Cases that need to be averted in the first year alone[c] | 14.2 | 7.2 | 18.5 |
| One-way sensitivity analysis | | | |
| Increasing labor and equipment costs from $20,000 to $50,000 | | | |
| Cases that need to be averted every year for 5 years[b] | 6.4 | 3.1 | 8.6 |
| Cases that need to be averted in the first year alone[c] | 20.9 | 10.6 | 27.2 |
| Decreasing the costs of an infection from $7,788 to $3,894 | | | |
| Cases that need to be averted every year for 5 years[b] | 9.9 | 4.8 | 13.2 |
| Cases that need to be averted in the first year alone[c] | 28.4 | 14.5 | 36.9 |
| Increasing the probability of death from 0.4% to 2.3% | | | |
| Cases that need to be averted every year for 5 years[b] | 1.5 | 0.5 | 2.6 |
| Cases that need to be averted in the first year alone[c] | 4.3 | 1.5 | 7.3 |

[a]The most frequently assumed discount rate is 5%. However, 3% is the recommended rate. No discounting is suggested for testing the sensitivity of the results (10).

[b]Threshold number of cases averted every year for 5 years above which the system is cost-beneficial.

[c]Threshold number of cases averted in the first year above which the system is cost-beneficial, assuming the system does not avert any cases in subsequent years and continues to incur costs.

beneficial. Unproductive extensive traceback investigations of sporadic ***E. coli*** O157:H7 infections have been conducted (12). Investigating such sporadic cases can be very costly (Table 1), and a subtype-specific system can reduce such costs.

According to the National Electronic Telecommunications System for Surveillance, 90 cases of ***E. coli*** O157:H7 were reported in Colorado in 1998 (13), an annual incidence rate of 2.3 per 100,000 population. In comparison, the national incidence rate calculated from these data was 1.2 per 100,000 population (3,161 cases).

This study was limited by lack of data that would have enabled us to estimate attack rates from the outbreak, cases averted by the meat recall, and the benefit to society (money saved) by establishing the system. Despite its limitations, this study has important implications for public health policy. From a societal perspective, a surveillance system does not need to prevent a large number of cases to yield return on the resources invested in it.

### Acknowledgments

The authors thank Patrick McConnon, Paul Mead, Peter Drotman, and Pam Shillam for helpful discussions.

This research is supported in part by CDC and Oak Ridge Institute of Science and Education through an interagency agreement between the US Department of Energy and CDC.

Dr. Elbasha is a health economist in the Prevention Effectiveness Branch, Division of Prevention Research and Analytic Methods, Epidemiology Program Office, CDC. His research interests include assessment of the burden of diseases, the cost-effectiveness and cost-benefits of various public health interventions, analysis of economic policies that impact public health, and integration of economic analysis and methods into epidemiologic models and data.

### References

1. Griffin PM, Tauxe RV. The epidemiology of infections caused by ***Escherichia coli*** O157:H7, other enterohemorrhagic ***E. coli***, and the associated hemolytic uremic syndrome. Epidemiol Rev 1991;13:60-98.
2. American Gastroenterological Association. Consensus statement: ***Escherichia coli*** O157:H7 infections: an emerging national health crisis, July 11-13, 1994. Gastroenterology 1995;108:1923-34.
3. Roberts T, Buzby J, Lin J, Mead P, Nunnery P, Tarr PI. Economic aspects of ***E. coli*** O157:H7: disease outcome trees, risk, uncertainty, and social cost of disease estimates. In: Prediction, detection, and management of tomorrow's epidemics. Greenwood B, De Cock K, eds. John Wiley & Sons, Chichester, UK. pp 156-72.
4. Buzby JC, Roberts T, Lin JC-T, MacDonald JM. Bacterial foodborne disease: medical costs and productivity losses. Washington: U.S. Department of Agriculture, Economic Research Service. AER No. 741. August 1996.
5. Centers for Disease Control and Prevention. Preliminary report: foodborne outbreak of ***Escherichia coli*** O157:H7 infections from hamburgers—western United States, 1993. MMWR Morb Mortal Wkly Rep 1993;42:85-6.
6. Bender JB, Hedberg CW, Besser JM, Boxrud DJ, MacDonald KL, Osterholm MT. Surveillance for ***Escherichia coli*** O157:H7 infections in Minnesota by molecular subtyping. N Engl J Med 1997;337:388-94.
7. Kolata G. Detective work and science reveal a new lethal bacteria. New York Times. 1998 Jan 6;147:A1, A14.
8. Bell BP, Goldoft M, Griffin PM, Davis MA, Gordon DC, Tarr PI, et al. A multiple outbreak of ***Escherichia coli*** O157:H7-associated bloody diarrhea and hemolytic uremic syndrome from hamburgers: the Washington State experience. JAMA 1994;272:1349-53.
9. Haddix AC, Teutsch SM, Shaffer PA, Dunet DO, editors. A guide to decision analysis and economic evaluation. New York: Oxford University Press; 1996.
10. Gold MR, Siegel JE, Russell LB, Weinstein MC, editors. Cost-effectiveness in health and medicine. New York: Oxford University Press; 1996.
11. Landefeld JS, Seskin EP. The economic value of life: linking theory to practice. Am J Public Health 1982;6:555-66.
12. Centers for Disease Control and Prevention. Enhanced detection of sporadic ***Escherichia coli*** O157:H7 infections—New Jersey, July, 1994. MMWR Morb Mortal Wkly Rep 1993;44:417-8.
13. Centers for Disease Control and Prevention. Summary of notifiable diseases, United States, 1998. MMWR Morb Mortal Wkly Rep 1999;47:1-93.

## References

[PDF_00350] Bell B. P., Goldoft M., Griffin P. M., Davis M. A., Gordon D. C., Tarr P. I., Bartleson C. A., Lewis J. H., Barrett T. J., Wells J. G. (1994). A multistate outbreak of Escherichia coli O157:H7-associated bloody diarrhea and hemolytic uremic syndrome from hamburgers. The Washington experience.. JAMA.

[PDF_00344] Bender J. B., Hedberg C. W., Besser J. M., Boxrud D. J., MacDonald K. L., Osterholm M. T. (1997). Surveillance by molecular subtype for Escherichia coli O157:H7 infections in Minnesota by molecular subtyping.. N Engl J Med.

[PDF_00364] Centers for Disease Control and Prevention (CDC) (1995). Enhanced detection of sporadic Escherichia coli O157:H7 infections--New Jersey, July 1994.. MMWR Morb Mortal Wkly Rep.

[PDF_00321] Griffin P. M., Tauxe R. V. (1991). The epidemiology of infections caused by Escherichia coli O157:H7, other enterohemorrhagic E. coli, and the associated hemolytic uremic syndrome.. Epidemiol Rev.

[PDF_00361] Landefeld J. S., Seskin E. P. (1982). The economic value of life: linking theory to practice.. Am J Public Health.

